# Estradiol Treatment Enhances Behavioral and Molecular Changes Induced by Repetitive Trigeminal Activation in a Rat Model of Migraine

**DOI:** 10.3390/biomedicines10123175

**Published:** 2022-12-08

**Authors:** Eleonóra Spekker, Zsuzsanna Bohár, Annamária Fejes-Szabó, Mónika Szűcs, László Vécsei, Árpád Párdutz

**Affiliations:** 1 ELKH-SZTE Neuroscience Research Group, University of Szeged, Semmelweis u. 6, H-6725 Szeged, Hungary; 2 Department of Medical Physics and Informatics, University of Szeged, Korányi Fasor 9, H-6720 Szeged, Hungary; 3 Department of Neurology, Interdisciplinary Excellence Centre, Albert Szent-Györgyi Medical School, University of Szeged, H-6725 Szeged, Hungary

**Keywords:** primary headache, migraine, trigeminal system, CGRP, nNOS, neurogenic inflammation, animal model, inflammatory soup, dura mater, estrogen, behavior

## Abstract

A migraine is a neurological condition that can cause multiple symptoms. It is up to three times more common in women than men, thus, estrogen may play an important role in the appearance attacks. Its exact pathomechanism is still unknown; however, the activation and sensitization of the trigeminal system play an essential role. We aimed to use an animal model, which would better illustrate the process of repeated episodic migraine attacks to reveal possible new mechanisms of trigeminal pain chronification. Twenty male (M) and forty ovariectomized (OVX) female adult rats were used for our experiment. Male rats were divided into two groups (M + SIF, M + IS), while female rats were divided into four groups (OVX + SIF, OVX + IS, OVX + E2 + SIF, OVX + E2 + IS); half of the female rats received capsules filled with cholesterol (OVX + SIF, OVX + IS), while the other half received a 1:1 mixture of cholesterol and 17β-estradiol (OVX + E2 + SIF, OVX + E2 + IS). The animals received synthetic interstitial fluid (SIF) (M + SIF, OVX + SIF, OVX + E2 + SIF) or inflammatory soup (IS) (M + IS, OVX + IS, OVX + E2 + IS) treatment on the dural surface through a cannula for three consecutive days each week (12 times in total). Behavior tests and immunostainings were performed. After IS application, a significant decrease was observed in the pain threshold in the M + IS (0.001 < *p* < 0.5), OVX + IS (0.01 < *p* < 0.05), and OVX + E2 + IS (0.001 < *p* < 0.05) groups compared to the control groups (M + SIF; OVX + SIF, OVX + E2 + SIF). The locomotor activity of the rats was lower in the IS treated groups (M + IS, 0.01 < *p* < 0.05; OVX + IS, *p* < 0.05; OVX + E2 + IS, 0.001 < *p* < 0.05), and these animals spent more time in the dark room (M + IS, *p* < 0.05; OVX + IS, 0.01 < *p* < 0.05; OVX + E2 + IS, 0.001 < *p* < 0.01). We found a significant difference between M + IS and OVX + E2 + IS groups (*p* < 0.05) in the behavior tests. Furthermore, IS increased the area covered by calcitonin gene-related peptide (CGRP) immunoreactive (IR) fibers (M + IS, *p* < 0.01; OVX + IS, *p* < 0.01; OVX + E2 + IS, *p* < 0.001) and the number of neuronal nitric oxide synthase (nNOS) IR cells (M + IS, 0.001< *p* < 0.05; OVX + IS, 0.01 < *p* < 0.05; OVX + E2 + IS, 0.001 < *p* < 0.05) in the caudal trigeminal nucleus (TNC). There was no difference between M + IS and OVX + IS groups; however, the area was covered by CGRP IR fibers (0.01 < *p* < 0.05) and the number of nNOS IR cells was significantly higher in the OVX + E2 + IS (*p* < 0.05) group than the other two IS- (M + IS, OVX + IS) treated animals. Overall, repeated administration of IS triggers activation and sensitization processes and develops nociceptive behavior changes. CGRP and nNOS levels increased significantly in the TNC after IS treatments, and moreover, pain thresholds and locomotor activity decreased with the development of photophobia. In our model, stable high estradiol levels proved to be pronociceptive. Thus, repeated trigeminal activation causes marked behavioral changes, which is more prominent in rats treated with estradiol, also reflected by the expression of the sensitization markers of the trigeminal system.

## 1. Introduction

Migraine is a primary headache causing throbbing pain and neurological symptoms such as photophobia, phonophobia, cutaneous allodynia, and decreased physical activity [1]. The disease is three times more common in women than men [2], therefore, the gonadal hormones may play a role in nociceptive processing and the development of migraine attacks. In addition, the increase in the frequency of migraine attacks is one important risk factor contributing to migraine becomes chronic [3].

A widely-used rodent model of migraine is the use of inflammatory soup (IS) [4,5,6,7], which reproduces the characteristic features of clinical migraine—both the structural [8] and functional [9] changes. The administration of IS onto the dura mater of the rat can activate the trigeminal system and induces a sterile inflammation [9,10]. Furthermore, it leads to cutaneous mechanical and thermal allodynia [11,12]. Based on these the IS model can be a valuable platform for preclinical studies. However, the current migraine models mostly use single stimulation [12,13], which does not take into account the prolonged and recurrent nature of attacks, which occur usually more than once within a month. With an exception to one study [7], repeated IS application in other models are usually continuous, which does not reflect the characteristics of repeated attacks [14,15]. Therefore, using an animal model in which discontinuous multiple stimulations are carried out on the dura mater would be more in line with recurrent episodic migraine attacks and helps to understand the mechanism of trigeminal pain becoming chronic.

In addition to studying molecular variances, it is crucial to examine behavioral changes as well in this context. The symptoms of migraine, e.g., the headache, the sensitivity to light and sound, and the allodynia are aggravated by physical activity [1,16]. Some experiments suggest that the application of inflammatory mediators directly onto the dura mater elicited tactile allodynia [17,18]. Moreover, in animal models of migraine, the locomotor activity of the animals was decreased [16,19], and photo- and phono-phobia were observed [20,21].

In animals, upon activation of the trigeminal system, neuropeptides are released, such as calcitonin gene-related peptide (CGRP), which has a vital role in migraine [22]. Repetitive electrical stimulation of the dura mater can increase CGRP expression in the trigeminal system [23]. Its release from trigeminal nerve endings may mediate the inflammation, contributing to the associated pain [24]. It has been also shown that CGRP may play an important role in the development of mechanical allodynia, hyperalgesia [25], and photophobia [26].

Some studies suggest that nitric oxide (NO) has a role in the transmission of inflammation [27,28] and can implicate the development of chronic pain [29]. The production of NO is catalyzed by nitric oxide synthase (NOS), and the neuronal isoform is expressed all along the migraine pain pathway, including the dura and pia mater [30]. An association has been demonstrated between the activation of the trigeminovascular system with the production or upregulation of nNOS. of the activation of the trigeminovascular system with the production or upregulation of nNOS [30], and the inhibition of NOS attenuates inflammatory pain [31,32]. Handy and colleagues described that after intraplantar injection of carrageenan, NOS inhibitors could prevent the hyperalgesia in response to a thermal or mechanical stimulus [33].

In our experiment, we used multiple IS stimulations onto the dura mater, based on the appearance of repeated episodic migraine attacks which is defined as less than 15 headache days per month with an attack duration of 4 to 72 h [1]. For mimicking multiple attacks, the rats received twelve IS treatments on three consecutive days for four weeks.

Among the gonadal steroids, mainly estradiol can modify the clinical appearance of migraine. After puberty, migraine occurs two to three times more often in women than in men, and the underlying processes behind the gender dimorphism are not yet known. Estrogen can elicit a pronociceptive effect by activating trigeminal afferents, enhancing glutamatergic tone, and increasing the levels of brain-derived neurotrophic factor (BDNF) or nerve growth factor (NGF) [34]. Based on these, it can be assumed that estrogen plays an important role in migraine attacks, therefore, we examined the effect of chronic, stable high-serum 17β-estradiol levels on trigeminal pain and activation.

Behavioral tests were also performed to examine the pain threshold, locomotor activity, and photophobia of the animals in these models. Furthermore, CGRP and nNOS immunohistochemical stainings were carried out and evaluated based on the somatotopic organization of the trigeminal nerve, which helped to examine the effect of the chemical stimulation of dura in a more specific way.

Our aim was to investigate how repeated IS treatment on the dura mater affects the behavior of the animals and the expression of activation and sensitization markers in TNC. Moreover, we wanted test the effect of a constant high estradiol level in this context, comparing ovariectomized females treated with estradiol to untreated male rats.

## 2. Materials and Methods

### 2.1. Animals

The procedures used in our study were approved by the Committee of the Animal Research of the University of Szeged (I-74-6/2020) and the Scientific Ethics Committee for Animal Research of the Protection of Animals Advisory Board (XI./1995/2020). They followed the guidelines the Use of Animals in Research of the International Association for the Study of Pain and the directive of the European Parliament (2010/63/EU).

Twenty male and forty female Sprague-Dawley rats weighing 280–350 g were used. The animals were raised and maintained under standard laboratory conditions with tap water and regular rat chow available ad libitum on a 12-h dark,12 h-light cycle.

### 2.2. Brief Summary of the Experiment

At week 0, female animals were ovariectomized and capsules were placed subcutaneously. In week 1, behavioral tests were performed with the animals to obtain baseline values. The following day, a craniotomy was carried out and a cannula was inserted into the animals’ skulls. The next week, before the treatments, we repeated the behavioral tests, meaning we checked whether the surgery caused any changes in the animals’ behavior. The next day we started the treatments. The animals were treated with SIF or IS for 3 consecutive days a week for 4 weeks, and various behavioral tests were performed one h after the treatment. At the end of the week 5 the animals were perfused, and samples were collected. A detailed description of the solutions and methodologies can be found below.

### 2.3. Experimental Groups

M + SIF: male rats with SIF treatment

M + IS: male rats with IS treatment

OVX + SIF: ovariectomized female + cholesterol capsules + SIF treatment

OVX + IS: ovariectomized female + cholesterol capsules + IS treatment

OVX + E2 + SIF: ovariectomized female + a 1:1 mixture of cholesterol and 17β-estradiol capsules + SIF treatment

OVX + E2 + IS: ovariectomized female + a 1:1 mixture of cholesterol and 17β-estradiol capsules + IS treatment

### 2.4. Ovariectomy

Forty female Sprague Dawley rats were used. The animals were ovariectomized under isoflurane anesthesia (Tec3 Selectatec Vaporizers, Harvard Apparatus, Holliston, MA, USA). Rats were put in a plastic box as an induction chamber that received a continuous flow of anesthetic gas. During induction, the rats were monitored by observing their respiratory movements and the pink color of their skin. After 10 min in the induction chamber (using induction doses of 5% isoflurane) the rat was quickly removed, and an inhalation mask was applied to it for the maintenance of anesthesia (using 3% isoflurane) during the surgical procedure.

Before surgery, the backs of the rats were shaved with an electric clipper to remove the fur, and Cutasept was used to disinfect the skin. First, a midline dorsal skin incision was made, which was 3 cm long and located approximately halfway between the middle of the back and the base of the tail, and then 1.5 cm-long peritoneal incisions were made on both sides. After access into the peritoneal cavity, the ovarian fat pad was carefully pulled out of the incision. With the help of hemostatic tweezers, the part below the ovary was tightly fixed. Thereafter, two knots were tied under the area to be removed using sterile thread, and then the connection between the fallopian tube and the uterine horn was cut and the ovaries were removed. Afterward, the peritoneal incisions were sutured together with sterile thread. Then, the animals were randomly divided into two groups. (1) In the OVX group, the rats had two 15 mm long silastic capsules (3.18 mm outer diameter and 1.57 mm inner diameter, catalog ID: 508–008; Dow Corning, Midland, MI, USA) filled with cholesterol (15 mg, catalog ID:C8667; Sigma-Aldrich, Darmstadt, Germany) as the control. (2) In the OVX + E2 group, the animals received two 15 mm-long silastic capsules filled with a 1:1 mixture of 17β-estradiol (7.5 mg, catalog ID: 75262; Fluka, Sigma-Aldrich) and cholesterol (7.5 mg), which provide a constant elevated serum estradiol level.

**Figure 1:**
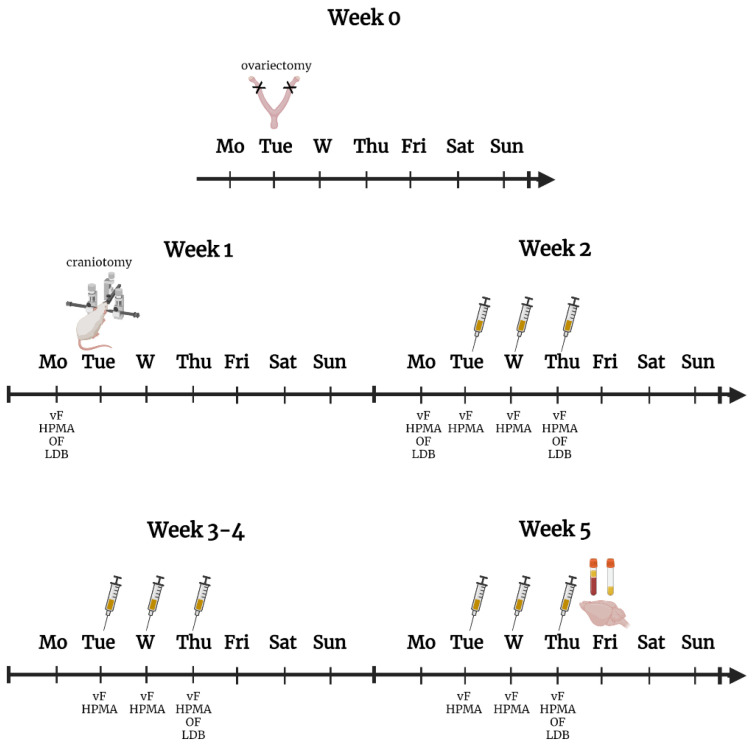
Schematic timeline of the experimental settings. One week before craniotomy, the female rats were overiectomized. After craniotomy, animals received twelve times SIF or IS treatment (needles). Behavioral tests were performed one day before and six days after the craniotomy and on the days when animals received treatment. After the twelfth SIF or IS treatment, the animals were transcardially perfused, and the trigemino-cervical complex was removed for immunohistochemistry. vF—von Frey test, HPMA—hind paw mechanical allodynia test, OF—open field test, LDB—light-dark box test. Created with BioRender.com.

The capsules were placed subcutaneously in the interscapular region, and then the peritoneal cavity and skin were closed with absorbable sutures. All the surgical instruments were sterilized in 70% ethanol. A high degree of aseptic procedure was followed during the procedure with all equipment being sterilized before. To prevent hypothermia, the animals were placed on a warmed pad (30–35 °C) and covered with paper. To relieve pain and avoid inflammation, the animals were injected subcutaneously with carprofen (5 mg/kg body weight) before surgery and 24 and 48 h after surgery. All animals had a six-day recovery period before the craniotomy (Figure 1).

### 2.5. Implantation of the Cannula

Both male and female animals were deeply anesthetized with an intraperitoneal injection of 4% chloral hydrate (0.4 g/kg body weight, Sigma-Aldrich). The head of the animal was fixed in a stereotaxic frame and lidocaine infiltration (4.5 mg/kg; subcutaneously) on the skull was used before the interventions. An incision was performed to expose the surface of the skull. The bone surface was then treated with 10% hydrogen peroxide to clean the wound and decrease the bleeding. The craniotomy (1 mm in diameter) was performed with an electric drill. The hole, located on the right side of the midline, 1 mm to the junction of the coronal suture and midline was drilled to expose the dura mater. To avoid burning and damage to the dura, a standard saline solution was used to decrease the temperature. After this, stainless steel cannula was made from a 21 G needle [35] and was affixed to the bone around the opening in the skull using small screws and two types of dental cements (Duracryl Plus, Adhesor Zinc Phosphate Cement, Spofa Dental, CZK, Jičín). The cannula’s end opened onto the dura and was sealed with an obturator that extends just beyond the end of the cannula over the dura. This prevented scar tissue from growing over the hole. At the end of the craniotomy, the animals received subcutaneous injections of carprofen (5 mg/mL, 0.1 mL/100 g, Pfizer, New York, NY, USA) and gentamicin (0.2 mL/100 g, Pfizer), which were repeated on the next two days after surgery (24 and 48 h). All animals had a six-day recovery period before the actual dural stimulation (Figure 1).

### 2.6. Application of the Inflammatory Soup or Synthetic Interstitial Fluid

The animals were randomly divided into two groups. Rats were placed in a transparent plastic box, where the treatment was administered to them. The animals in the first group, called the control group, received synthetic interstitial fluid (SIF, 135 mM NaCl, 5 mM KCl, 1 mM MgCl_2_, 5 mM CaCl_2_, 10 mM glucose, in 10 mM HEPES buffer, pH 7.3) as treatment. In the second group, we applied inflammatory soup (IS, 1 mM bradykinin, 1 mM serotonin, 1 mM histamine, 0.1 mM prostaglandin in 10 mM HEPES buffer) on the dural surface. The solutions (10 µL) were delivered to the surface of the dura mater through a cannula made of polyethylene tube (PE10) and a 30 G needle manually, using a Hamilton syringe, over 5 min while the rat was freely moving (Figure 1). During the experiment, the animals received a total of 12 treatments (three consecutive days for 4 weeks).

Time points for repeated inflammatory soup treatment were chosen based on the headache criteria. In episodic migraine, the frequency of headache days is less than 15 days per month, and the attacks usually last from 4 to 72 h. Based on these, rats were infused with IS or SIF for three consecutive days per a week to better model the symptoms of migraine patients. With the repeated IS treatment, we mimicked multiple attacks.

### 2.7. Behavioral Tests

The behavioral tests were performed the day before the craniotomy and the first treatment, as well as on the treatment days one hour after SIF or IS administration (Figure 1).

#### 2.7.1. von Frey Test (vF)

The testing procedures were performed during the light phase (between 8 a.m. and 2 p.m.) in a quiet room. Three weeks before the experiment, the rats were habituated to the von Frey filament to get used to touching the area between the eyes with the fibers. The rodents were placed in a transparent plastic box, which prevented the rat from walking away from the sensory testing, but it was large enough for the animals to turn around with some difficulty. During the test, von Frey filaments, identified by manufacturer-assigned force values (Bio-VF-M von Frey Filaments, Bioseb, USA, Florida), were used to touch the area between the two eyes and observe the reaction of the animals. We touched the rats five times in a row with a filament. There was a small 2-min break between the five touches. If the animal jerked its head at least three times out of five touches, the test was positive and over for the animal. If it did not respond to that filament, we used another filament with higher strength (Figure 1).

#### 2.7.2. Hind Paw Mechanical Allodynia Test (HPMA)

Ten minutes after the von Frey test, a hind paw mechanical allodynia test was performed. We used a dynamic plantar aesthesiometer (37450; Ugo Basile, Gemonio, Italy) to measure the touch sensitivity threshold in both hind paws. The device presses the paws of the animals with a maximum force of 50 g, rising continuously from 0 and reaches a maximum of 50 g in 8 s from touching the sole. When the animal showed a paw-withdrawal response, the value of g printed by the machine was recorded. We pressed both feet three times, and then the test was over. During the statistical analysis, we used the mean of these three measurements (Figure 1).

#### 2.7.3. Open Field Test (OF)

An open field test can be used to measure spontaneous locomotor activity. The animals were placed in an open box from above (48 × 48 × 40 cm); then, during the 5 min of the experiment, the time spent moving or grooming and the time spent in the center of the box were detected with a video camera (Basler GigE acA1300-60 gm Ahrensburg, Germany, Ethovision XT 14 software. Noldus Information Technology, Wageningen, The Netherlands) (Figure 1).

#### 2.7.4. Light Dark Box Test (LDB)

This test can be used to determine the anxiety response in rodents and is also suitable for studying photophobia. The animals were placed in a special box (Experimetria Ltd. Hungary, Budapest), which has a dark and a light side. The light compartment is 2/3 of the box and is brightly lit and open. The dark compartment is 1/3 of the total box and is covered and dark. A door of 7 cm connects the two compartments. At the beginning of the test, the animals were placed in the light area. After 5 s, the door opened, then they could go through the dark side. After ten minutes, the test ended, and the animals were removed from the box. The test examined anxiety and photophobia based on how much time the animals spent in the light and dark areas (Figure 1).

During IS treatments, we monitored the animals on a daily basis. We kept track of the changes by scoring the weight change, appearance, posture, ability to move, and respiration. If any given animal reached a critical score, we excluded it from the experiment.

### 2.8. Measurement of Estradiol Concentration

The serum 17β-estradiol concentration was measured in both groups (*n* = 5). At the end of the experiment, blood samples were taken from the female rats. The serum was cleared from cellular components of the blood by centrifugation at 12,000 rpm for 10 min at 4 °C and stored at −80 °C until use. An Estradiol EIA Kit (catalog ID: 582251; Cayman Chemical Company, Ann Arbor, MI, USA) was used to assay concentrations according to the guidelines of the manufacturer.

### 2.9. Immunohistochemistry

At the end of the experiment, the anesthetized animals (4% chloral hydrate) were trancardially perfused (50 mL PBS, 0.1 M, pH 7.4), then fixed with 200 mL 4% paraformaldehyde in phosphate buffer. Afterward, the trigemino-cervical complex was removed, post-fixed overnight in the same fixative., and then processed for CGRP and nNOS immunohistochemical staining. After cryoprotection (10% sucrose for 2 h, 20% sucrose until it sank, and 30% sucrose for 1 night) 30 µm sections were made using a cryostat and serially collected in 12 wells containing cold PBS.

Thirty serials of sections were collected into 10 wells starting from one millimeter rostrally to the obex. Every tenth section was used for staining. The free-floating sections were rinsed in PBS and immersed in 0.3% H_2_O_2_ in methanol or PBS for 30 min. After several rinses in PBS containing 1% Triton X-100, sections were kept overnight at room temperature in the anti-CGRP antibody (Sigma-Aldrich, Darmstadt, Germany, C8198) at a dilution of 1:20,000, or for two nights at 4 °C in the anti-nNOS antibody (EuroProxima, 2263B220-1, Arnhem, Netherlands) at a dilution of 1:5000. The immunohistochemical reaction was visualized by the avidin-biotin kit (Vectastain, Vector Laboratories, Newark, CA, USA, PK6101) and 3,3′-diaminobenzidine enhanced with nickel ammonium sulfate. The specificity of the immune reaction was controlled by omitting the primary antiserum.

### 2.10. Data Evaluation

All evaluations were performed by an observer blind to the procedure. The photomicrographs of the CGRP and nNOS stained sections were taken using a Zeiss AxioImager M2 microscope supplied with an AxioCam MRc Rev. 3 camera (Carl Zeiss Microscopy, New York, NY, USA) with a 10_x_ and 40_x_ objective with Zeiss Zen Pro 2.6 software. The area covered by CGRP-IR fibers was determined by Image Pro Plus 6.2^®^ image analysis software (Media Cybernetics, USA, MD, Rockville). After image acquisition, the laminae I–II in the dorsal horn were defined manually as areas of interest, and a threshold gray level was validated with the image analysis software. The program calculated the area innervated by the IR fibers as the number of pixels with densities above the threshold; the data were expressed as area fractions (%) of the corresponding immunolabelled structures. The nNOS-IR cells were counted in laminae I-II of the dorsal horn using Nikon Optiphot-2 light microscope (Nikon, Tokyo, Japan) under 10× objective. We measured the covered area by the CGRP-IR fibers and counted the nNOS-IR cells according to the somatotopic representation of the ophthalmic (V/1) branch (Strassman and Vos, 1993).

### 2.11. Statistical Analysis

Prior to our experiments, we employed the PS Power and Sample Size program to determine the number of animals required. The Shapiro–Wilk test was used to determine the distribution of data. In addition, we used a Q-Q plot to find out if two sets of data come from the same distribution. Our data followed a normal distribution. For CGRP and nNOS immunohistochemistry the differences among the groups and sides were examined with a mixed ANOVA model. The effects of treatments on nNOS cell numbers and the area covered by CGRP-IR fibers at various distances from the obex between the treated and untreated sides were examined with distance, treatment, and side as repeated measures (within-subject factor) and the group as between-subject factors. RM ANOVA test was used to evaluate the results of the behavior tests followed by the Tamhane post hoc test. For the 17β-estradiol concentration of serum paired and independent samples *t*-tests performed the pairwise comparisons with Sidak corrections. All statistical analyses was carried out using SPSS version 24.0 (IBM Corporation, New York, NY, USA). Values *p* < 0.05 were considered statistically significant. Our data are reported as means + SEM for all parameters and groups.

## 3. Results

### 3.1. Estradiol Concentration

**Figure 2:**
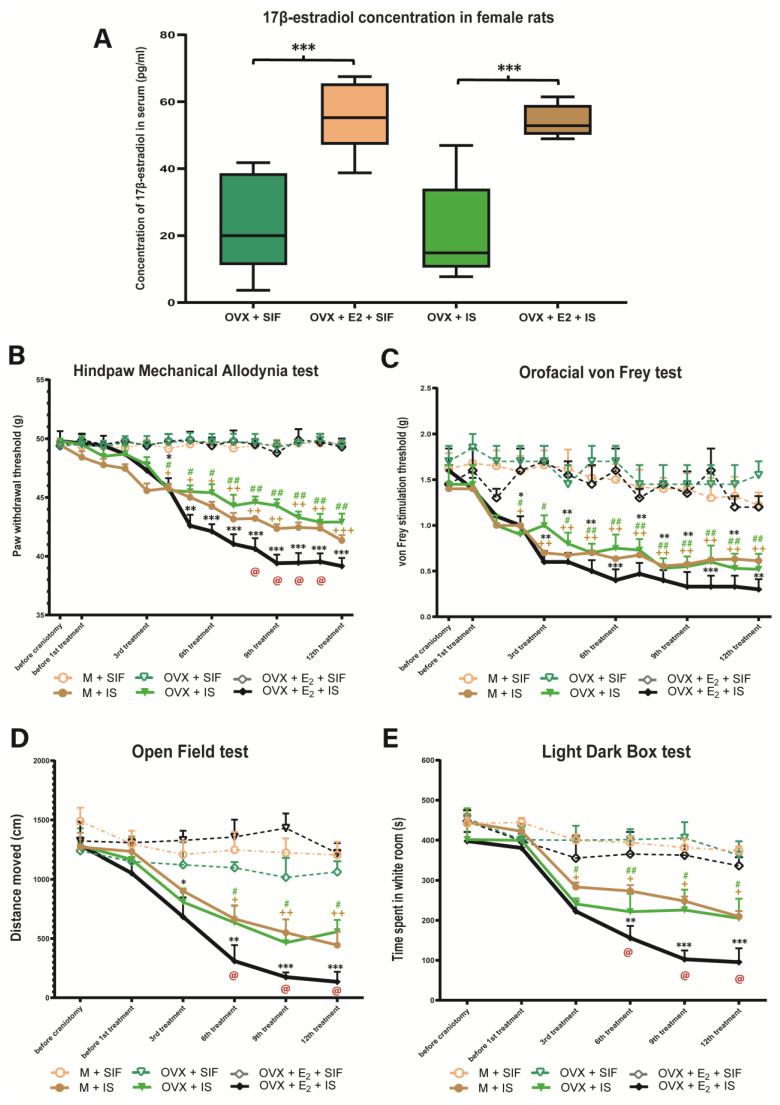
Statistical analysis of the serum concentration of 17-estradiol and behavior tests. (**A**) The concentration of 17β-estradiol in serum (pg/mL) in the female rats. The chronic 17β-estradiol treatment significantly increases the serum concentration compared with the cholesterol (*** *p* < 0.001). (**B**) Hind Paw Mechanical Allodynia test. The IS treatment significantly decreased the paw withdrawal threshold. In the OVX + E2 + IS group this change was even more significant. (**C**) Orofacial von Frey test. After the second IS administration, the pain threshold was significantly lower in OVX + IS, OVX + E2 + IS and M + IS treated groups compared to the control groups. There was no difference between male and female animals. (**D**) Open Field test. The distance moved by rats was significantly less from the third treatment in the IS treated groups compared to the control groups and 17β-estradiol treatment further reduced this. (**E**) Light Dark box test. The animals in the IS treated groups spent more time in the dark room compared to the control groups. Chronic estrogen treatment reduced the time spent in the light compared to the male rats. + means M + SIF and M + IS, # means OVX + SIF and OVX + IS, and * means OVX + E2 + SIF and OVX + E2 + IS difference. @ means M + IS and OVX + E2 + IS difference. (* *p* in the range from 0.05 to 0.01; ** *p* in the range of 0.01 to 0.001; *** *p* < 0.0001).

The ovariectomy kept an approximate steady-state status in serum concentration of 17β-estradiol was maintained for 6 weeks with an average value of 20.96 pg/mL in the OVX and OVX + IS group, and 54.05 pg/mL in the OVX + E2 and OVX + E2 + IS group (Figure 2A).

### 3.2. Inflammatory Soup and Behavioral Changes

There was no difference in the paw withdrawal threshold between the groups before and after the craniotomy, but a significant decrease was detected in the pain threshold after the fourth IS treatment in the hind paw mechanical allodynia test. Chronic estrogen treatment further reduced the pain threshold compared to the male rats. There was a significant difference between the OVX + E2 + IS and M + IS groups. The OVX + IS group was similar to the M + IS group (Figure 2B).

Similar to the previous behavior tests, before IS treatment, there was no relevant difference between the control and the treated groups in the orofacial von Frey test, but after the second IS infusion, the pain threshold was significantly lower in OVX + IS, OVX + E2 + IS and M + IS treated groups compared to the control groups. There was no significant difference in male and female rats (Figure 2C).

Prior to the cannulation, no noticeable difference in locomotor activities was observed between the control and the treated groups, but the distance moved by rats was significantly decreased from the third treatment in the IS-treated groups compared to the control groups. Similar to HPMA test, in female animals given IS in addition to chronic estradiol treatment, an even greater decrease is observed in the distance traveled compared to male animals (Figure 2D).

In the light-dark box test, the animals of the IS-treated groups spent more time in the dark room compared to the control groups, but in the number of passes there was no difference between the two groups. Chronic estrogen treatment reduced the time spent in the light compared to the male rats. There was a significant difference between the OVX + E2 + IS and M + IS groups. The OVX + IS group was similar to the M + IS group (Figure 2E).

### 3.3. Inflammatory Soup and Calcitonin Gene-Related Peptide

**Figure 3:**
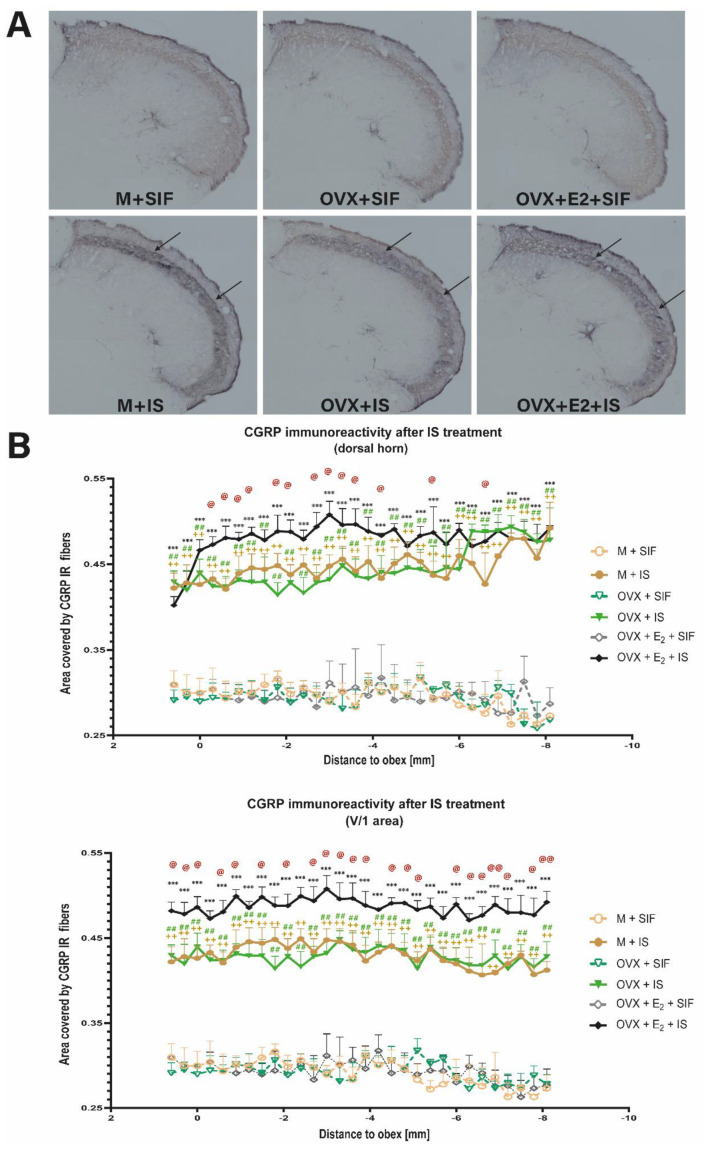
CGRP immunostaining. (**A**) Representative photomicrographs of the CGRP expression in the trigemino-cervical segments. In the IS treated groups, the CGRP staining was stronger compared to the control groups. (**B**) Statistical analysis of CGRP staining. In the IS-treated groups, the area covered by CGRP-IR fibers is significantly higher than in the control groups in the whole dorsal horn and in the ophthalmic nerve area. + means M + SIF and M + IS, # means OVX + SIF and OVX + IS, and * means OVX + E2 + SIF and OVX + E2 + IS difference. @ means M + IS and OVX + E2 + IS difference. (* *p* in the range from 0.05 to 0.01; ** *p* in the range of 0.01 to 0.001; *** *p* < 0.0001).

After CGRP staining of the caudal trigeminal nucleus, CGRP-IR axon fibers were distributed in the laminae I and II in the dorsal horn. In the IS treated groups, the CGRP staining was stronger than in the control groups (Figure 3A). There was a significant increase in the area covered by CGRP IR fibers in the IS-treated groups in the ophthalmic nerve area. In the case of the maxillary nerve and mandibular nerve, the difference is negligible. Higher CGRP immunoreactivity was observed in female animals receiving chronic estrogen treatment and IS compared to the male IS-treated rats. No difference was observed between the OVX + IS and M + IS groups (Figure 3B).

### 3.4. Inflammatory Soup and Neuronal Nitric Oxide Synthase

**Figure 4:**
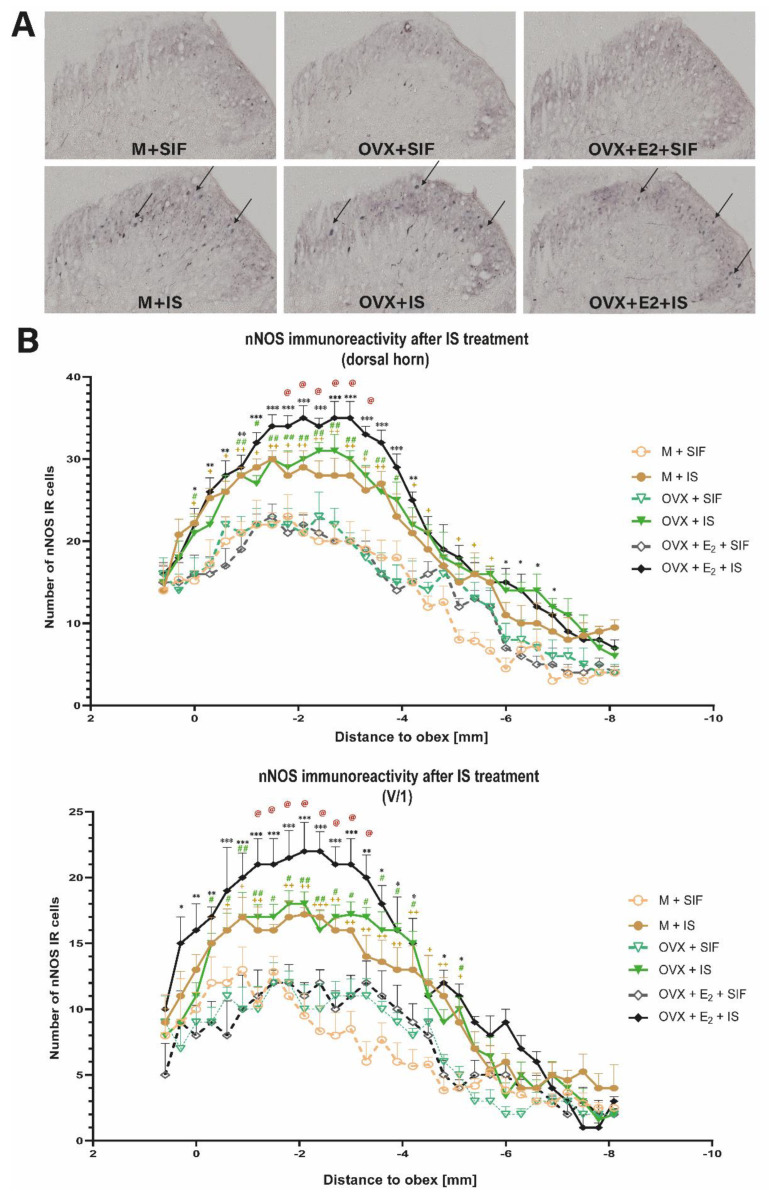
nNOS immunostaining. (**A**) Representative photomicrographs of the nNOS expression in the trigemino-cervical segments In the IS-treated groups, the nNOS staining was more robust than in the control groups. (**B**) Statistical analysis of nNOS staining. The quantitative analysis shows that in the IS-treated groups, the number of nNOS-IR cells was significantly higher than in the control groups in both areas. + means M + SIF and M + IS, # means OVX + SIF and OVX + IS, and * means OVX + E2 + SIF and OVX + E2 + IS difference. @ means M + IS and OVX + E2 + IS difference. (* *p* in the range from 0.05 to 0.01; ** *p* in the range of 0.01 to 0.001; *** *p* <0.0001).

Following the nNOS immunohistochemical staining of the TNC, more nNOS-positive cells were observed in the IS-treated groups than the control groups (Figure 4A). After the statistical analysis, the repeated administration of IS was able to increase the number of nNOS IR cells in the whole dorsal horn and the somatotopic representation of the ophthalmic nerve area. This difference is not seen for the maxillary and mandibular nerves. More nNOS-positive cells were found in female animals treated with chronic estrogen and inflammatory soup than in male IS-treated animals. There was no significant difference between the OVX + IS and M + IS groups (Figure 4B).

## 4. Discussion

Overall, in our experiment, the repeated administration of the IS was able to reduce the pain threshold and locomotor activity of the rats. In addition, the time spent in the light decreased in the group treated with IS, and the level of CGRP and nNOS increased in the TNC. Furthermore, estrogen treatment was able to further enhance the changes caused by IS, when compared to M + IS and OVX + IS groups.

The behavioral results showed that the infusion of IS leads to an increase in nociceptive responses. We found that the multiple administration of IS on the dura mater can cause a significant decrease in mechanical pain thresholds of both the face and hind paws in the orofacial von Frey test and the hind paw mechanical allodynia test (Figure 2B). Furthermore, we observed increased facial grooming and scratching behavior. These results may indicate pain and discomfort in the animals. During migraine attacks, many patients report cutaneous allodynia, which may be localized to the pain area of headache; however, the face and scalp may be affected as well as body and limbs [36]. In our experiment, throughout the orofacial von Frey, the pain threshold of the rats was significantly reduced (Figure 2C), which may suggest pain localized to the head area and cutaneous allodynia. The topical application of inflammatory soup to the dura mater could activate and sensitize Aδ and C fibers [5]. Multiple C fibers stimulations during repeated migraine attacks and the following activation of Aδ fibers lead to neurogenic inflammation in the trigeminovascular system [37]. As a result, hypersensitivity develops in the central trigeminal neurons, which results in an enhanced response to non-painful stimuli and an expansion of the receptive field [6]. Similar to the results obtained here, Edelmayer and colleagues described that allodynia induced by inflammatory mediators developed over several hours and was found not only on the face but also extrasegmentally on the hind paws [38]. Neuroinflammation leads to increased permeability of the blood–brain barrier, glial cell activation, and production of inflammatory mediators [39]. The activation of dorsal root ganglion neurons and microglia contributes to central sensitization [40]. The inflammatory processes and overexpression of CGRP nociceptive neurons are involved in the generation of pain hypersensitivity [41].

In addition to the decrease in the pain threshold, reduced locomotion was also observed in the open field test (Figure 2D), which might correspond to the reduced physical activity in patients with headaches because it increases the pain during migraine attacks. Worsening of migraine pain has often been perceived by patients during or after regular physical exercise [42]. In migraineurs, the throbbing pain can be aggravated by routine activities, therefore freezing and decreased locomotion might be a defense mechanism to limit the head and body movements of the animal. We hypothesized that animals experiencing a headache-like state would spend less time exploring the environment than those that are pain-free.

It should be noted that the decreased pain threshold and locomotor activity became more pronounced throughout the experiment. The behavioral changes we observed can be paralleled with human data, e.g., patients with a long history of more frequent and severe headaches may be more likely to develop sensitization and have allodynia. Bigal and colleagues described that cutaneous allodynia is more common and more severe in migraine than in other primary headaches and it is associated with the headache frequency, increased body mass index, disability, and depression [43]. Moreover, there is evidence of a direct and increasing correlation between allodynia and the duration of migraine [44]. Similar results were obtained by Louter and colleagues, namely, that there is an association between cutaneous allodynia and the number of migraine days [45]. This relationship may be explained by the repetitive activation of modulatory pain pathways. Another concept is that a noxious stimulus may lead to a sensitized state and chronification of pain. Due to the increasingly frequent attacks, the time between the attacks is decreasing, so the threshold may not be able to return to baseline. In addition, sensitization processes may further lower the threshold.

Furthermore, we found that in the IS treated group, the animals spent less time in the light part of the light–dark box (Figure 2E). This may also be related to the photophobia experienced in migraine sufferers. A study showed, that in rats the light is able to activate specifically dural-nociceptive posterior thalamic neurons, and this activation increases with increasing light intensity [46]. The processing of craniovascular nociceptive information in posterior lateral posterior and dorsal thalamic neurons relay directly to cortical areas, suggesting a role in cognitive and motor deficits during the migraine, as well as allodynia, photophobia, and phonophobia [47]. Vanagaite and colleagues found that migraineurs were more photophobic during the migraine attacks than outside the attacks, but even interictal migraine patients were more sensitive to light than controls [48]. In addition, Yalın et al. reported that a long duration of headache and a higher attack intensity correlate with more frequent incidences of nausea, vomiting, photophobia, and phonophobia [49].

Intracerebroventricular administration of CGRP causes a significant increase in light aversion, which can be prevented by the simultaneous administration of CGRP receptor antagonist, olcegepant [50].

In the trigeminal ganglia neurons, which transmit nociceptive signals from the head and face to the central nervous system, CGRP is expressed [51]. In migraine pathophysiology, CGRP is essential, especially in developing and maintaining chronic pain, and may be associated with allodynia and central sensitization [52]. An electrophysiological study described that CGRP, and its spinal receptors play a significant role in the generation and maintenance of the hyperexcitability of dorsal horn neurons caused by inflammation [53]. Greco et al. described that the level of CGRP was significantly higher in chronic migraine, or a medication-overuse headache compared to episodic migraine. Besides that, CGRP levels correlate with monthly migraine days [54]. Cernuda-Morollón and colleagues showed an increased CGRP serum level in chronic migraine compared to episodic migraine, thus CGRP may play a role in migraine chronification [55].

In our experiment, IS elevated the area covered by CGRP-IR fibers in the dorsal horn and ophthalmic nerve area (Figure 3).

Overall, these results suggest that CGRP may play a part at both the central and peripheral level in migraine mechanisms and promote the hypothesis that migraine patients have a combination of changed sensory perception of not harmful stimuli and altered brainstem and trigeminovascular activation.

In the central nervous system, CGRP and nNOS interact and may dilate meningeal vessels, presumably by the NO-inducing CGRP release of sensory fibers, resulting in vessel dilation [56]. In the central nervous system, NO plays a crucial role in central pain sensation and mediates neurotransmission and is involved in inflammatory response [57]. The activation of primary afferent neurons and CGRP release may induce nNOS by NO [58]. Berger and colleagues have shown that nNOS is present in nerve fibers of the dura mater in rats [59] and in the trigeminal nerve endings and in the TNC and the trigeminal ganglion [60]. Pradhan et al. described that chronic intermittent injection of NTG can cause an acute and chronic hypersensitivity that persists for days after the last exposure. These results are in line with clinical observations of patients with chronic migraine in whom allodynia may occur both between and during migraine attacks [61].

In our study, IS significantly increased the number of nNOS IR cells both in the whole dorsal horn and the somatotopic area of the ophthalmic nerve, responsible for the somatosensory innervation of the dura (Figure 4) [62]. This is in line with what has been described previously that nitroglycerin administration can increase the NOS-immunoreactivity in dura mater [63]. However, after three IS treatment, there was a significant difference between the two groups for the maxillary and mandibular branches as well. This can be explained by the fact, that NOS related NO diffuses freely across membranes rapidly and exert its activity in a more widespread way, not necessarily following the somatotopy, at least after three IS treatments. Thus, NO can activate signaling cascades both within the cell in which it was produced and by freely passing through membranes and activating nearby cells [30]. The increased nNOS expression in second-order trigeminal nociceptors might initiate a self-amplifying process of NO production at the basis of central sensitization [64]. Probably, the production of NO, via the increased expression of nNOS, mediates the production and release of CGRP and pituitary adenylyl-cyclase activating polypeptide as well [30]. Thus, nNOS can be considered a significant marker of the trigeminal system sensitization process in repeated episodic trigeminal activation.

In our experiment, chronic estradiol treatment significantly decreased the pain threshold, the locomotor activity and the time spent in the light (Figure 2). Since the results of male and OVX none treated animals are similar it seems valid to state that the key factor in this experimental setting is the presence or absence of estradiol. These findings are consistent with previous results, where estradiol exposure was able to increase allodynia [65,66] and the light aversion [20]. Similar results were obtained in other experimental models [67,68,69]. In the orofacial formalin model, after chronic estrogen treatment, the number of c-FOS IR cells in the TNC and the pain-related behavior of the animals increased, thus the high estrogen level had a pronociceptive effect in our experiment [70]. In another model, where inflammation was induced in the masseter muscle, a single treatment with estradiol-valerate increased facial allodynia [65,66]. In the model of temporomandibular joint inflammation, several days of estradiol treatment increased the inflammatory processes and reduced the appetite of the animals [71]. Others found that orofacial pain and thermal hyperalgesia induced by s.c. carrageenan injection were exacerbated by 17β-estradiol treatment [72]. Estrogen receptors are present in areas important for trigeminal nociception, including the trigeminal ganglion and TNC [73,74]. It is conceivable that estrogen may influence pain-induced neuronal processes through an increase in transient receptor potential vanilloid mRNA. 1 and anoctamine 1 in TNC. Furthermore, it cannot be ruled out that it exerts its effect by modulating the NF-κB pathway or by activating ERK in the trigeminal ganglion.

In addition to behavioral differences, we also observed that in the dorsal horn, the area covered by CGRP IR fibers, and the number of nNOS IR cells was significantly higher in chronic estrogen-treated animals compared to M + IS and OVX + IS groups (Figure 3 and Figure 4).

All estrogen receptors are expressed in different parts of the trigeminovascular system, such as the rodent trigeminal ganglion [73,74] and dura mater [20]. Previous studies described that estrogen could positively enhance the expression of CGRP within the dorsal root ganglion [75,76,77,78], rat anterior pituitary [79], and medial preoptic nucleus [80]. Estrogen pretreatment increased dural mast cell density, suggesting that gonadal steroids can modify CGRP function [81].

García-Durán and colleagues reported that during ovulation the expression of nNOS protein in neutrophils was higher than in the follicular phase, suggesting that there may exist an association between the level of estrogen and nNOS expression in neutrophils [82]. Furthermore, Ceccatelli and colleagues described that in ovariectomized rats, the estradiol treatment increased the nNOS mRNA in the ventrolateral subdivision of the ventromedial nucleus in female rats [83].

Unfortunately, our experiment has some limitations. On one hand, our model can only mimic partial aspects of the migraine attacks, in this case, the neurogenic inflammation. On the other hand, we used animals with stable gonadal hormone levels to test the effect of estradiol, whereas normally, the female gonadal hormone levels are changing. Further experiments with cycling female animals are needed to better understand the gender difference.

## 5. Conclusions

Overall, in our experiment, multiple administrations of IS was able to activate and sensitize the trigeminal system and develop nociceptive behavior changes. Decreases in pain threshold and locomotor activity and the development of photophobia occurred in our experiment. Moreover, CGRP and nNOS levels increased significantly in the TNC due to the IS treatments. We found a difference between male and female animals, which reinforces the role of estrogen in migraine attacks. Based on these findings, our method may be suitable for modeling repeated episodic migraine.

## Data Availability

The data presented in this study are available on request from the corresponding author.

## Abbreviations

CGRP	calcitonin gene-related peptide
E2	17β-estradiol
HPMA	hind paw mechanical allodynia test
IR	immunoreactive
IS	inflammatory soup
LDB	light-dark box test
NO	nitric oxide
NOS	nitric oxide synthase
NTG	nitroglycerin
OF	open field test
OVX	ovariectomized
PBS	phosphate-buffered saline
PBS-T	PBS containing 1% Triton-X-100
PFA	paraformaldehyde
SIF	synthetic interstitial fluid
TNC	transient receptor potential ankyrin 1
vF	von Frey test
